# Supplementary material for: SyNDI: synchronous network data integration framework
Source: BMC Bioinformatics. 2018 Nov 6;19:403. doi: 10.1186/s12859-018-2426-5 (PMC6219086; doi:10.1186/s12859-018-2426-5)
Supplement: Supplementary file 5 — Files of the example presented in Additional file 4. This additional file is a zip package that contains all files used in Additional file 4. Detailed descriptions of each of these files are presented in a README file included in this zip package. (GZ 30411 kb) [file 12859_2018_2426_MOESM5_ESM.gz › SyNDITools/Meme2Fimo/meme_4.9.0_4.tar.gz!/meme_4.9.0/website/html/gomo-intro.html]

GOMO - Introduction


|  |  |
| --- | --- |
|  | MEME Suite The MEME Suite web application requires the use of JavaScript   Javascript doesn't seem to be available on your browser.  GOMO -- Gene Ontology for MOtifs  **The purpose of GOMO is to identify possible roles (Gene Ontology terms) for DNA binding motifs.**  GOMO returns a list of GO-terms that are significantly associated with target genes of the motif, sorted by *q*-value (minimum false discovery rate). Gene Ontology provides a controlled vocabulary to describe gene and gene product attributes in any organism.   GOMO takes a motif and scores the upstream (promoter) region of each gene in the selected organism according to its binding affinity for the motif. Using these scores and a the GO annotations of the organism's genes, GOMO determines which GO terms are associated with the (putative) target genes of the binding motif. GOMO reports the *p*-value for each GO-term as well as the *q*-value, which adjusts the *p*-value for multiple tests. The *q*-value is defined as the minimal false discovery rate at which a given GO-term occurrence is deemed significant. GOMO determines *q*-values using the method of Benjamini and Hochberg. GOMO reports all GO terms with *q*-values smaller than the threshold specified by the user.  By default, for some organisms, GOMO also scores the upstream (promoter) regions of all orthologous genes in a set of related genomes, and uses these scores to improve the sensitivity of the search for GO terms associated with the motif. The organisms for which the GOMO website supports this comparative genomics mode can be seen by clicking on the **supported database** link on the GOMO submission form. To score upstream regions, the GOMO web service uses the average binding affinity score, which computes the average likelihood ratio of all positions in a sequence. This score is computed using the AMA MEME Suite tool. |
